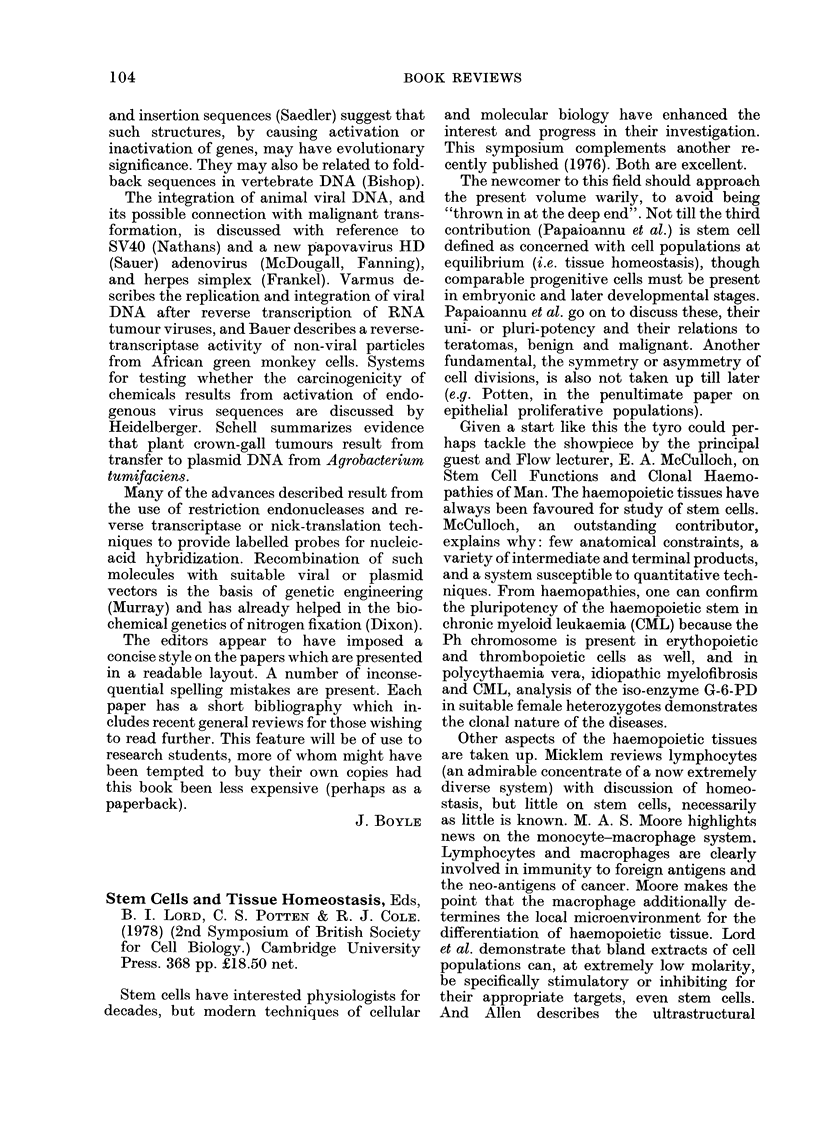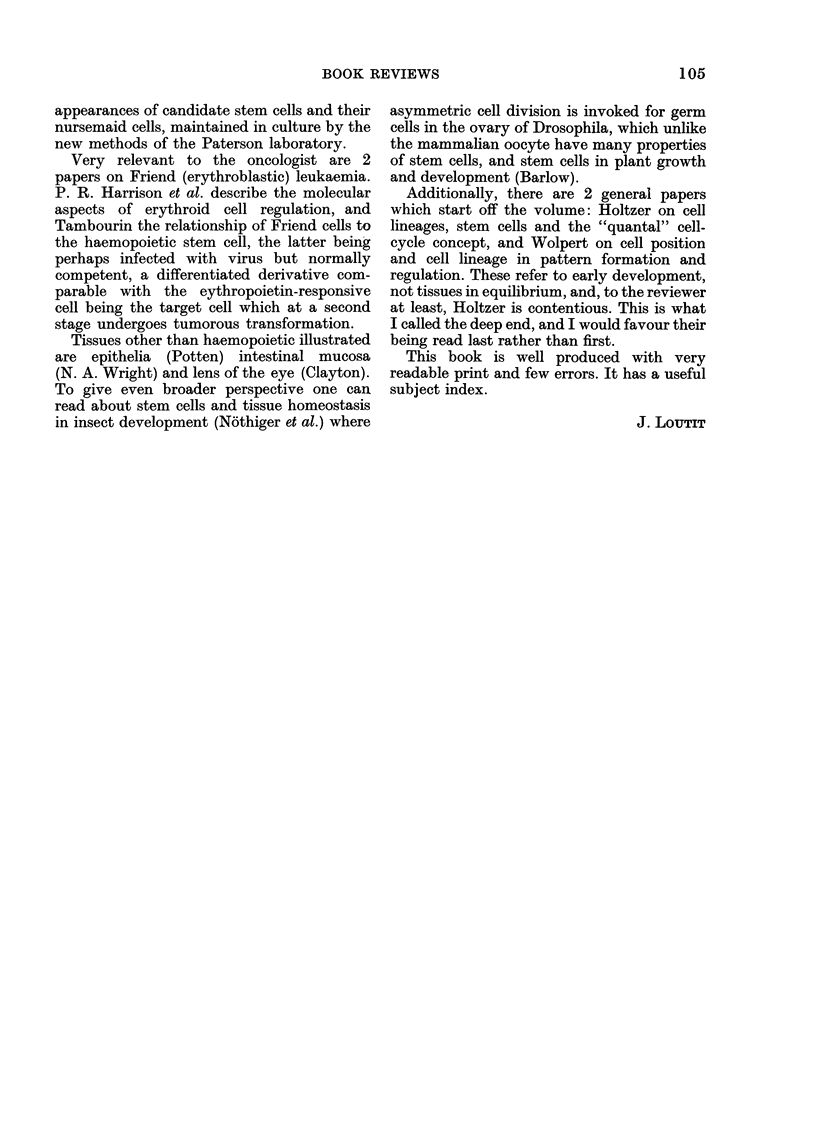# Stem Cells and Tissue Homeostasis

**Published:** 1979-01

**Authors:** J. Loutit


**Stem Cells and Tissue Homeostasis,** Eds, B. I. Lord, C. S. Potten & R. J. Cole. (1978) (2nd Symposium of British Society for Cell Biology.) Cambridge University Press. 368 pp. £18.50 net.

Stem cells have interested physiologists for decades, but modern techniques of cellular and molecular biology have enhanced the interest and progress in their investigation. This symposium complements another recently published (1976). Both are excellent.

The newcomer to this field should approach the present volume warily, to avoid being "thrown in at the deep end". Not till the third contribution (Papaioannu *et al.*) is stem cell defined as concerned with cell populations at equilibrium (*i.e.* tissue homeostasis), though comparable progenitive cells must be present in embryonic and later developmental stages. Papaioannu *et al.* go on to discuss these, their uni- or pluri-potency and their relations to teratomas, benign and malignant. Another fundamental, the symmetry or asymmetry of cell divisions, is also not taken up till later (*e.g.* Potten, in the penultimate paper on epithelial proliferative populations).

Given a start like this the tyro could perhaps tackle the showpiece by the principal guest and Flow lecturer, E. A. McCulloch, on Stem Cell Functions and Clonal Haemopathies of Man. The haemopoietic tissues have always been favoured for study of stem cells. McCulloch, an outstanding contributor, explains why: few anatomical constraints, a variety of intermediate and terminal products, and a system susceptible to quantitative techniques. From haemopathies, one can confirm the pluripotency of the haemopoietic stem in chronic myeloid leukaemia (CML) because the Ph chromosome is present in erythopoietic and thrombopoietic cells as well, and in polycythaemia vera, idiopathic myelofibrosis and CML, analysis of the iso-enzyme G-6-PD in suitable female heterozygotes demonstrates the clonal nature of the diseases.

Other aspects of the haemopoietic tissues are taken up. Micklem reviews lymphocytes (an admirable concentrate of a now extremely diverse system) with discussion of homeostasis, but little on stem cells, necessarily as little is known. M. A. S. Moore highlights news on the monocyte–macrophage system. Lymphocytes and macrophages are clearly involved in immunity to foreign antigens and the neo-antigens of cancer. Moore makes the point that the macrophage additionally determines the local microenvironment for the differentiation of haemopoietic tissue. Lord *et al.* demonstrate that bland extracts of cell populations can, at extremely low molarity, be specifically stimulatory or inhibiting for their appropriate targets, even stem cells. And Allen describes the ultrastructural appearances of candidate stem cells and their nursemaid cells, maintained in culture by the new methods of the Paterson laboratory.

Very relevant to the oncologist are 2 papers on Friend (erythroblastic) leukaemia. P. R. Harrison *et al.* describe the molecular aspects of erythroid cell regulation, and Tambourin the relationship of Friend cells to the haemopoietic stem cell, the latter being perhaps infected with virus but normally competent, a differentiated derivative comparable with the eythropoietin-responsive cell being the target cell which at a second stage undergoes tumorous transformation.

Tissues other than haemopoietic illustrated are epithelia (Potten) intestinal mucosa (N. A. Wright) and lens of the eye (Clayton). To give even broader perspective one can read about stem cells and tissue homeostasis in insect development (Nöthiger *et al.*) where asymmetric cell division is invoked for germ cells in the ovary of Drosophila, which unlike the mammalian oocyte have many properties of stem cells, and stem cells in plant growth and development (Barlow).

Additionally, there are 2 general papers which start off the volume: Holtzer on cell lineages, stem cells and the "quantal" cell-cycle concept, and Wolpert on cell position and cell lineage in pattern formation and regulation. These refer to early development, not tissues in equilibrium, and, to the reviewer at least, Holtzer is contentious. This is what I called the deep end, and I would favour their being read last rather than first.

This book is well produced with very readable print and few errors. It has a useful subject index.

J. Loutit